# Experience of Health Leadership in Partnering With University-Based Researchers in Canada– A Call to "Re-imagine" Research

**DOI:** 10.15171/ijhpm.2019.66

**Published:** 2019-08-07

**Authors:** Sarah Bowen, Ingrid Botting, Ian D. Graham, Martha MacLeod, Danielle de Moissac, Karen Harlos, Bernard Leduc, Catherine Ulrich, Janet Knox

**Affiliations:** ^1^Applied Research and Evaluation Consultant, Centreville, NS, Canada.; ^2^Department of Community Health Sciences, University of Manitoba Winnipeg, Winnipeg, MB, Canada.; ^3^Ottawa Research Institute, University of Ottawa, Ottawa, ON, Canada.; ^4^School of Nursing, University of Northern British Columbia, Prince George, BC, Canada.; ^5^Universite de Saint-Boniface, Winnipeg, MB, Canada.; ^6^University of Winnipeg, Winnipeg, MB, Canada.; ^7^Hôpital Montfort, University of Ottawa, Ottawa, ON, Canada.; ^8^Northern Health, Prince George, BC, Canada.; ^9^University of Northern British Columbia, Prince George, BC, Canada.; ^10^Nova Scotia Health Authority, Halifax, NS, Canada.

**Keywords:** Research Partnerships, Integrated Knowledge Translation, Health Systems, Canada, Health System Leadership

## Abstract

**Background:** Emerging evidence that meaningful relationships with knowledge users are a key predictor of research use has led to promotion of partnership approaches to health research. However, little is known about health system experiences of collaborations with university-based researchers, particularly with research partnerships in the area of health system design and health service organization. The purpose of the study was to explore the experience and perspectives of senior health managers in health service organizations, with health organization-university research partnerships.

**Methods:** In-depth, semi-structured interviews (n = 25) were conducted with senior health personnel across Canada to explore their perspectives on health system research; experiences with health organization-university research partnerships; challenges to partnership research; and suggested actions for improving engagement with knowledge users and promoting research utilization. Participants, recruited from organizations with regional responsibilities, were responsible for system-wide planning and support functions.

**Results:** Research is often experienced as unhelpful or irrelevant to decision-making by many within the system. Research, quality improvement (QI) and evaluation are often viewed as separate activities and coordinated by different responsibility areas. Perspectives of senior managers on barriers to partnership differed from those identified in the literature: organizational stress and restructuring, and limitations in readiness of researchers to work in the fast-paced healthcare environment, were identified as major barriers. Although the need for strong executive leadership was emphasized, "multi-system action" is needed for effective partnerships.

**Conclusion:** Common approaches to research and knowledge translation are often not appropriate for addressing issues of health service design and health services organization. Nor is the research community providing expertise to many important activities that the healthcare system is taking to improve health services. A radical rethinking of how we prepare health service researchers; position research within the health system; and fund research activities and infrastructure is needed if the potential benefits of research are to be achieved. Lack of response to health system needs may contribute to research and ‘evidence-informed’ practice being further marginalized from healthcare operations. Interventions to address barriers must respond to the perspectives and experience of health leadership.

## Background

There is increasing recognition of the potential benefits of participatory research to address the many and complex problems currently facing healthcare systems across the world.^1-7^ Collaboration in health research may take many forms.^8,9^ In addition to interdisciplinary partnerships between researchers and research teams, there is increasing emphasis on partnerships between researchers and the *intended users or beneficiaries* of the research (patients, local communities, clinicians, policy-makers, or health system leaders and managers). The many traditions of partnered research (action-oriented research, co-production of knowledge, integrated Knowledge Translation, Mode 2 research, engaged scholarship)^10^ share the common characteristics of meaningful engagement of stakeholders or potential research knowledge users from the beginning of the research process, and selection of research questions of importance to them.^11,12^

### Proposed Benefits of Health System-Researcher Partnerships

Interest in researcher-knowledge user collaboration in health research has been driven, in large part, by recognition of the lack of relevance, applicability and utility of much academic research.^13-16^ There is emerging evidence that meaningful knowledge user engagement is a major predictor of research utilization.^17-22^ Although there is limited evidence on the ultimate impacts of researcher/health system partnership on system functioning,^22-26^ potential benefits identified include: improved quality of solutions,^27^ greater research relevance and credibility,^28^ enhanced capacity of both researchers and knowledge users,^29^ greater understanding of partners’ roles,^28^ personal and professional development,^29^ greater likelihood that research will be applied in practice,^30^ and spin-off benefits such as enhanced skills and networks for future activities.^28,29,31^

Research on potential benefits of knowledge co-production in the health sector has attained prominence at the same time that critiques of earlier approaches to promoting knowledge use have emerged. These approaches, based on simple linear and often uni-directional models of knowledge transfer,^32-34^ and strategies to bridge the gap between the supposed different ‘cultures’ of research partners,^34-36^ have had limited impact. This awareness, along with interest in measuring the impacts of research investments,^37^ has led to many major health research funders requiring participation by health system leadership in health research.^38-41^ As a result, researchers are increasingly required to find a health system partner, and health system personnel are often approached to partner with academic (university-based) researchers.

### Knowledge Gaps

In contrast to what we have learned about challenges to, and facilitators of, *research use* by decision-makers,^19^ minimal attention has been given to challenges and facilitators affecting *research co-production.* Additionally, based on our review of the literature, most research has focused on partnerships between clinicians or policy-makers (eg, government officials), and (in recent years) on patient engagement in research, rather than on perspectives and experiences of health system leaders (eg, senior management of health delivery organizations). Few resources are available for health leadership on selecting, establishing and managing effective research partnerships,^24,42-43^ although recent research has proposed ‘guiding principles,’ ‘mechanisms,’ or ‘features’ of effective collaboration.^23,25,44-47^ This lack of practical guidance is of particular concern in the field of health services and health systems research, where co-production has been defined as an ‘essential dimension.’^48^

Much research on partnerships is based on assumptions of researcher-driven initiatives,^12^ and often fails to include perspectives of health system leaders.^49,50^ Researchers and decision-makers may have different experiences of the same processes.^51,52^ Despite support of health system management for the principles of collaborative research,^44^ emerging reports of actual experiences often highlight challenges.^18,44,53,54^ There is often evidence of lack of genuine collaboration by many academic researchers.^23,44,55^ In addition, many research requests for partnership are to address narrowly focused research questions: little is known about research partnerships that address issues of system design and health service organization,^56,57^ the focus of our research.

This paper summarizes findings from interviews with senior health personnel across Canada, one activity of the research project “*Building and Managing Effective Partnerships in Canadian Health Research* ” (https://iktrn.ohri.ca/). The purpose of these interviews was to explore the experience and perspectives of health system leadership (ie, senior managers based in health service organizations with responsibility for health service delivery) with health organization-university research partnerships. This work was supported by the Canadian Institutes of Health Research (CIHR) funded Foundation Grant (FDN #142337) “*Moving Knowledge into Action for more effective practice, programs and policy: A research program focusing on integrated knowledge.* ”

Canada has a publicly funded health system consisting of 13 provincial and territorial healthcare insurance plans.^58^ With the exception of a few smaller provinces/territories, responsibility for health delivery has been moved from the governmental department to regionally-based bodies that plan and deliver publicly funded services at the local level. These bodies (eg, health authorities) are generally responsible for the funding and delivery of hospital, community, and long-term care, as well as mental and public health services. Organization of health services differs significantly between provinces and territories, and regionally-based health organizations are diverse in structure and size.^59^ Over the past several years, many provinces have restructured their health delivery systems, with resulting changes in both the mandate and size of these delivery bodies. In some cases, service delivery functions have been separated from planning and coordinating functions.

## Methods

The research team consisted of 6 researchers with organizational, health services, engaged scholarship, and knowledge translation expertise, and skill in both qualitative and quantitative methods. Some brought experience in working within the health system, including in research-related roles. One member was bilingual (French/English). This national team recruited 4 senior level health executives as advisors to provide guidance to the research – helping ensure appropriate research questions, and providing additional insights in data interpretation.

A list of all health regions in Canada was compiled (n = 64) based on their configuration in 2018. We defined “health region” to include regional health authorities, health regions, provincial health delivery bodies, local health integration networks in Ontario and the Integrated Health and Social Service Centres and Integrated University Health and Social Services Centres of Quebec. We focused our research on health services organization at the regional level rather than on specific institutions or sites. While institutional bodies often are active in bench or clinical research, they are generally not directly involved in research on health system design and health service organization.

An interview guide including both open and closed-ended questions was designed to address the following research questions:

- How is research defined and understood within Canadian health regions?
- To what extent, and in what ways, is research used in organizational planning/innovation?
- What challenges are experienced by the health system in accessing, initiating, and managing research that is useful to them in planning around health system design and organization?
- What strategies are used to initiate and manage effective partnerships with academic researchers?
- What suggestions do health leaders have for creating effective partnerships that will address priority health system issues?

Consent forms and recruitment materials were translated into French: contact with Quebec regions (where French is the official language) was made in French, and interviews conducted by a Francophone researcher. The majority of interviews were conducted by SB and IB, both of whom were experienced interviewers and had worked within research support/coordination roles within regional health organizations. DdM conducted the French language interviews.

### Participants and Sampling

A purposive sampling strategy was used, focusing on identifying individuals within regions who had an active leadership role in research partnerships and held support and coordination functions across the system (eg, as Director of Research, or Director of Planning). We felt that individuals in such roles would be best positioned to make thoughtful observations on the experience and perspectives of senior health management (from executive level to the next level below, typically Director and Manager) within their region. The initial sample (n = 17) was drawn from a list of senior personnel identified through a review of regional websites conducted in preparation for this study; representatives of regions suggested by team members based on known collaborative research activity; and others who had expressed interest in the project (eg, in response to presentations on earlier research at national conferences). Snowball sampling, and efforts to identify individuals from under-represented areas of Canada, supplemented this initial list (n = 18). All potential participants were given an identification number at the time they were identified.

Potential participants were contacted by email, and provided with a copy of the consent form and links to additional project information. A reminder email was sent after 2 weeks, with one additional reminder sent if needed.

### Analysis

Interviews were audio-recorded and transcribed verbatim: French language interviews were audio-recorded, and transcribed directly into English by the bilingual interviewer. Two researchers (SB and IB) analyzed the transcripts independently, using a two-stage process. Directed content analysis^60^ was used for the first phase of analysis: researchers began by using key concepts as initial coding variables (eg, partnership challenges). This was followed by coding on additional themes arising during the interviews. Differences in interpretation between the 2 researchers were discussed, with extensive discussion of the appropriate emphasis to be given to emerging themes.

An internal report, based on initial coding and identification of themes, was circulated to the full research team. This extensive report, which included large numbers of de-identified quotes extracted from the transcripts, was the basis of a full-day research team meeting to further query the data and discuss data interpretation. Additional actions to help ensure trustworthiness included: consulting project Advisory Network members about emerging findings; and distribution to all participants of a summary report, with request for feedback. This informant feedback (“member checking”) phase was used to verify that conclusions made resonated with participant experience.^61^

## Results

### Participation

Twenty-five participants completed in-depth semi-structured telephone interviews between June and November 2018. Fourteen (82%) of those approached in the first round of invitations completed an interview, and 11 (61%) of those invited in the second round, for an overall response rate of 73%. Many of those who responded indicated their interest in the project, and stressed the importance of the research question. Of the 10 who did not complete an interview, 2 required provincial ethical review/application for a research license before participating; 2 declined; one was on leave, and one suggested another individual from the same region. The remainder did not respond to initial or follow up invitations. Interviews ranged from 30 minutes to over one hour (average 50 minutes). Nineteen of the 25 participants held a position within a health region, and had a role in research or research partnerships. Of these, all had cross-program system-wide responsibility (eg, regional research portfolio or responsibility for regional planning), although 4 had more focused roles in acute care, and 3 in primary care. Thirteen were in director level roles or above (eg, chief executive officer [CEO], Vice President). The remainder held a variety of positions (eg, Research coordinator). Five had responsibility at the provincial/territorial level, either because they were located within a provincial department or because the province had only one health region. Three of the remaining 6 participants, all identified through snowball sampling, held senior positions in national organizations with a focused perspective on health quality and research; and 3 were from hospital-based research centres. Seventeen participants were female, 8 were male. Participants represented 9 of Canada’s 13 provinces and territories; while medium-sized and smaller rural and northern regions were included, most were large health regions, often urban or provincial in scope. Several participants responded to the circulated report, indicating thanks and support for findings. Further interpretation with project advisors strengthened the findings: in some cases advisors expressed stronger critiques and greater frustration with current practice.

### Key Themes Emerging

Key themes emerging from this study are elaborated below under 3 main headings; Perspectives on Research and Its Usefulness; Organizational Experience with Research Partnerships; and Strategies for Research Partnerships: Health Leadership Perspectives.

### Perspectives on Research and its Usefulness

Many participants discussed the significant internal diversity in understanding and definitions of research within their organizations: “*[It] varies depending on the individual* ’*s background or experience, what they* ’*ve been exposed to in their career.* ” (Participant identification #35). While a few felt that research was broadly defined, many more felt that narrow definitions of research were common within their organization.

Lack of timeliness, narrowness of focus, and lack of skills in adapting research to a specific context often resulted in health leaders concluding that research was often not helpful to health system improvement efforts. As a result, as indicated by the quotes in Table 1, many participants observed that research was often viewed as marginal, or even not considered at all in organizational decision-making. As might be expected, therefore, the perceived importance of developing partnerships with academic researchers for the purpose of addressing questions of health system design and health system organization was also described in diverse ways. While most of those interviewed felt that such partnerships were very or extremely important, sometimes describing them as ‘*super-important* ’*(13),* ‘*paramount* ’ *(31),* ‘*vital* ’ *(19)* or ‘*critical* ’ *(26)* , this was in contrast to the importance they felt research partnerships were awarded within their organizations.

**Table 1:** Perspectives on Research and its Usefulness

**Key Themes**	**Sample Quotes**
Diversity of organizational understanding, perspectives on research	“*It is very variable. [Following amalgamation] leaders or directors coming from organizations that did not have a university [focus]... research is rather obscure* ” (13). “*I think in our region there would be the full range.There* ’*speople who would only see research as a purist, sort of objective kind ofendeavour, and there are others who would see it more broadly than that* …*. including the knowledge mobilization, knowledge exchange aspect* ” (17). “*Those leaders who still have academic connections totally get it...leaders who don* ’*t have that connection, and this is my perception only, they think research is the sort of old stereotype, disconnected from what my issues are* … *irrelevant to what they are doing. This underlying attitude exists across the vast majority of operations, I am going to say 80% plus* ” (05).
Prevalence of limited understandings and “definitions” of ‘research’	“*A lot of research is focused on more of the, I* ’*d say, gold standard and bench research. It* ’*s research that would be performed in a university, a very controlled design like an RCT* ” (18). “*People see research as being someone with a white coat and using chemicals and stuff like that. I don* ’*t think it* ’*s top of mind.* … *they* ’*re still focused on the little researcher in a lab doing things* ” (16). “*Gathering or creating evidence to support decision-making. There are still many* … *even leaders themselves, who would see that as not research, but would really define research by it being grant-funded, external funding* ” (23).
Limited extent that research is considered useful, particularly in organizational decision-making	“*The pace of change* … *research takes too much time; we need to do something now.* …*the move to complex adaptive systems* … *this is where organizations are moving - is not compatible with* ‘*research,* ’ *it is too rigid. There is a lack of awareness of different kinds of research; they are not interested if it is not tied to a grant. The pace of change, fiscal restraints, new regulations, research is not useful by the time you get it* ” (16). “…*you get a set of guidelines of something that* ’*s evidence-based or research-based and you look at it and you say, well I don* ’*t have this piece of equipment, I don* ’*t have this clinician.SoI guess we* ’*ll just keep doing what I* ’*ve always done, instead of that ability to contextualize those guidelines into an environment* ” (17). “*In decision-making, some will use it, others will forget to use it, so we aren* ’*t here. But* …*. it has been forgotten in [the structural transition] and we have to remind people that we have researchers with expertise from which we could benefit. But its baby steps, slowly* …” (11). “*Sometimes, I feel we are asked to come to the table to produce something helpful for a conversation, like the research part. That* ’*s helpful for the conversation, but it* ’*s almost just glanced at and not actually used in decision-making.Soit* ’*s almost like if it fits into the perspectives of the decision-makers who requested the research, then it would be considered. It might also be the persuasiveness of those who are at that table* …*. one person might feel really strongly that we need to go in a certain direction and it doesn* ’*t matter what the research says.* … *It definitely needs to fit the world view, it needs to fit what they feel is the political* …*, what the climate feels like at the time of the conversation* ” (18).
Low importance given to academic research partnerships	“*Critical to those leaders who understand research. Less important or not important to all those who are still in sort of the old culture of you know - academics are over there in their ivory towers not having a clue about anything* ” (05). “…*..I think the focus is on operations, it* ’*s getting people in the door and out the door* …*.There are VPs connected to the (universities) but for the specific purpose of saying we need you to help us solve a key problem in (the health authority), they* ’*re not doing that* ” (09). “*I* ’*m often the first one around a table to say* “*What universities or research institutes have done work in this area?* ” *or* “*Why don* ’*t we partner with someone to look at this?* ” *And I* ’*ll be looked at with this quizzical look - like why are you talking? Why should we partner with anyone?* ” (35). “*It* ’*s kind of neutral.* … *it* ’*s not something they see as part of their mandate. To develop greater partnerships with research, I just don* ’*t think it makes it to the top of the list* ” (29). “*Somewhat* – *I would probably say* ‘*very* ’ *[important] if I weren* ’*t a bit skeptical about the application of researcher experience to our jurisdiction* ” (04).
Lack of shared understandings, tension around concepts of research, and QI	“*I think there* ’*s a rub in our organization that has never really been reconciled, about the intersection between QIendeavoursand research* ” (17). “*A fine line between an improvement project and research* … *when there is potential for innovation, then it becomes research, there can be in the clinical setting a procedure that leads to an innovation* … *there is sharing of this information and the researcher can indicate his interest and fit it with his research...* ” (11). “*I don* ’*t think there* ’*s any value in trying to define what each of them is* … *the value is in what the purpose is and being clear about what we are trying to achieve* ” (05).
Focus on knowledge translation as communication of research findings	*“Uptake of information, even ifitsbeen well done, into service design, is very poor unlessitswithin the narrow scope of the (current) priorities”* (18). *“Often researchers, when they have done their research project, knowledge transfer stops at thepeer orientedcommunications and scientific meetings. But clinicians do not go to those meetings and do not read those scientific journals”* (13). *“We brought it to the executive and … I don’t think they really recognized what it really meant…. the knowledgetranslation specialistthen worked with the academic, but at the end of the day, the academic didn’t want to adjust (the report) to the health authority’s needs.Sothey came up with a document, it looks fantastic but I’m not sure it’s going to be used by our health authority.… I’m not sure that academics understand that, you know, you can’t sort of wave your magic wand and make things happen. And I was surprised that they didn’t want to be a bit more flexible…”* (09).

Abbreviations: QI, quality improvement; RCT, randomized controlled trial.

A key issue that emerged is how activities that are not formally funded research projects were viewed. In Canada, quality improvement (QI), evaluation and research activities are often seen as distinct, often have different sources of funding, and face different ethical review requirements. While some participants made clear distinctions between these 3 activities, others struggled with the ‘*fine line* ’ (11) or ‘*grey zone* ’ (31) or ‘*continuum* ’ (08) between them. Participants revealed that the 3 activities are often viewed as ‘belonging’ in different places: research with universities; QI within health organizations; and evaluation with external consultants. Evaluation was often not mentioned, and when discussed, was often viewed as distinct from both QI and research. Some identified neglect of evaluation in innovation and implementation as a problem. It was observed that researchers were often not interested in evaluation: even researchers employed by the health region may ‘*not see (evaluation) as research.* ’ It was noted that if those with research expertise were not involved in evaluation, “*you* ’*re losing therigourand scientific frameworks to do proper evaluation* ” (16). Both resistance to evaluation and limited views of evaluation (such as limiting it to outcome evaluation), were noted. Some noted that they found the distinctions between these 3 categories unhelpful, and a potential barrier to improved health system functioning.

While knowledge translation was only occasionally identified in the discussion of the definition of research, several participants referenced knowledge translation roles within their research portfolios and referred to challenges in promoting research utilization. Activities described focused most often on communicating research findings within the organization: the concept of co-creation of research was rarely addressed. Few examples were provided where regional staff was actively engaged in all stages of the research. Challenges in promoting research use, even when based on projects conducted within the organization, were described. Others expressed frustration either with the concept of knowledge translation itself or with the ‘knowledge transfer’ approach:

“… *when I hear there* ’*s 200 competencies around knowledge translation* … *seems like we* ’*re developing this whole complex lexicon to apply to things that have always been* … *sometimes I think the whole KT language sometimes muddies the water* ” (10).

### Organizational Experience With Research Partnerships

Several questions explored experiences with research partnerships, particularly partnerships addressing questions of health system design and health services organization. All participants recalled recent examples of partnerships: most were related to acute care, some to recent patient engagement initiatives. Fewer appeared to be related to health system change or service organization. While some participants described organizational actions to promote active involvement in research planning and decision-making, most described partnerships simply as an agreement to allow a project to be conducted within the organization, or to participate in research activities. Some expressed concerns about the quality of partnerships.

*“Collaborations on paper - I* ’*ve seen that a lot to be quite honest. Almost to the point where I say:* ‘*I* ’*m sorry, we can* ’*t provide a letter of support.* ’ *When you* ’*re asking for a letter of support and you* ’*re alluding to collaboration, what does that look like if you get funding? Because what will happen most times is the funding will come through and we* ’*ll never hear from them again”* (25).

*“I found over the years that people claim to have connections but they didn* ’*t hold a lot of water; connections were flimsy”* (07).

There was diversity in descriptions of how partnerships evolved: while most described a context where the topic emerged out of ongoing relationships, others stated that most partnerships were initiated by academics (“*the university, I would say more* ” (23)). Many described partnership relationships in generally positive terms, giving examples of specific relationships with individual academics that they had found helpful or positive. Others described the partnership relationship more neutrally, acknowledging both benefits and investments of time, skill and resources required for such relationships. Some highlighted the benefits of working with younger researchers, and felt that it helped researchers with their case for tenure.

*“We actually go for those young, up-and-coming researchers because they* ’*re just more prepared to do the work and not just ask for a letter of support but really, truly collaborate”* (25).

“… *young researchers who see the opportunity to work with us to develop their research program and ensure access to the health setting. Often that is what is at stake for researchers – to have access to the health setting* … *if he is already collaborating, and they have helped resolve problems and clinical issues, then the door is wide open”* (13).

### Challenges Experienced

Participants were asked specifically about any challenges they had experienced in partnerships in which they had been involved, and also to respond to a list of challenges identified in the literature (Supplementary file 1). Of all challenges about which they were questioned, only one was denied by the majority of participants: this was the issue of lack of organizational expertise to partner.

*“I would disagree with that. I think we do have the expertise to partner. I think we don* ’*t know everyone* ’*s expertise in academia; they haven* ’*t raised their hands and said they* ’*re interested. We don* ’*t know who or what they* ’*re like, who they are or what they might be doing or why they might be interested”* (18).

*“Infactwe had all the expertise, a lot of expertise in research and knowledge production and knowledge transfer, so we had the resources”* (13).

“*The health services people were much more astute about research than the university people gave them credit for”* (07).

In addition, although communication challenges were recognized and expected in professional interactions, some felt that academics and organizational staff did communicate, but they were not listening to each other: they were often having ‘*parallel conversations* ’ (18).

*“We* ’*re not really hearing what the other is saying and including that in our thinking.* … *if we suggest doing something slightly different than what they* ’*re granted to do, they don* ’*t even want to talk to us”* (18).

*“It* ’*s not that they don* ’*t communicate, it* ’*s the fact that they* ’*re communicating from different sets of assumptions* … *I think fundamentally it* ’*s a lack of understanding of what the differences are between their assumptions and how things need to be done* …*”* (23).

As indicated in Table 2, results did confirm findings of earlier studies that identified time demands of partnership research, timelines for action, and mismatch of researcher/decision-maker timelines as a major challenge.

**Table 2:** Challenges Experienced by the Health System

**Key Themes**	**Sample Quotes**
Time, timelines	*“When we ask a researcher to help develop a research project, he is in a cycle of funding agencies, where he has to build a project, find co-researchers, apply for grants, which are rejected, resubmitting for grants, so that takes 2-3 years sometimes…. what we see is much shorter for clinical improvement”* (13). *“My concern would be by the time you have the question, you set up the research and get the results, the system has already changed. The results we get will be a statement of what we were doing 12, 18 months ago. But when we get them, we’re not there anymore.Sowhat is the value of that? And when you have a lack of resources you need to be very mindful of where you are going to concentrate your energy”* (16). *“The biggest issue is the time constraints”* (22).
Health system restructuring	*“We reorganized research as well. Even in our research sector, we were caught between administration and scientific tasks in thecentres, restructuring, re-establishing processes... the fact that we haven’t focused yet on awareness and promotion of research …it’s not that we weren’t thinking about it, but don’t have time to do it and the resources to do it”* (13). *“Any time there’s kind of structural changes, it poses challenges, additional challenges to embedded and academic researchers.Soyou have to re-establish relationships maybe that you had already established”* (23). *“We’ve engaged and built awareness and put out ideas, but yeah, things are slow, especially when you’re faced with a lot more ‘important things’ like ‘transformation’”* (25). *“The provincial ministry … the issues were the same everywhere with the amalgamation. Research was forgotten”* (12).
Internal organizational change	*“When there’s a rework… when we reverted back to site management (from program management), we lost our connection with that level of leadership, and those were executive directors and directors and it’s been frustrating because sites think differently.Sothe structural changes have a huge impact”* (09). *“The way we set research priorities internally, it would make a greater difference because it’s mostly driven by what leadership would want us to focus on. And I think that’s where the hesitancy comes in when we start a project, and we’re kind of wondering, are we ever going to see the end of this?”* (18). *“Some of the areas that are under change in the health authority could impact ongoing research. Departments could be reorganized. People could no longer be there. Technology may change.… that’s also a challenge and I understand it’s very difficult for academic researchers who plan a program of research to find 6 months later when the grant is announced that some might have changed”* (31). *“A couple of changes in personnel or a couple of rounds of budget cutting can make it fall very quickly. So many organizations do great things, then the chief executive officer moves out anditcrumbles”* (34).
Health system stress	*“When there’s a lot of system change, just the culture moves away from innovation and striving towards excellence to more about survival, and managing crises - you know more of a scarcity model that doesn’t allow for possibility. One of the risks with that is that research becomes the domain of what happens in academic institutions or ivory towers … I wonder if some of the restructuring is sort of creating more of a pull-back, even though, like [federal health research funder] and other funding bodies tend to want to see more collaboration”* (25). *“In a large health authority, the imperative is the budget. And when you’re over budget, it’s very hard for decision-makers to free up time to think about are we doing the right thing?”* (09). *“The closer you get to the frontline in healthcare right now, the more frenetic it is.Sothere is a tendency for the urgency, immediacy of the decisions that are in front of people to overshadow the time that they might need to contemplate some things a little bit more deliberately with some attention to the evidence”* (17).
Costs of research to organization	*“I think in many projects we just list ‘in kind’ as if it’s going to happen without understanding what does ‘in kind’ mean, like ‘in kind’ suggests it’s over and above somebody’s current work, and in this environment, it’s very difficult”* (08). *“You have some studies where the data pull request has been unrealistic… they want records, like 30years worthof records for a certain kind of condition, you know, that involves both our analytic people and health records and that can’t be done for free”* (09). *“Often, researchers want the collaboration and would like to work with you, but they’re not prepared to plan a budget that pays for that contribution”* (21). *“We can’t apply directly for research support funds and … when we collaborate with academic researchers, the money usually goes to the university, so we don’t have a robust way of having discussions with our university partner. … I would say that has been a challenge especially when [federal research funding body] asks for cash contributions. That is when you are asking a health authority to empty out it’s pockets and we’re in savings mode all the time… That’s very, very difficult and that’s prohibitive to health research overall. …. I can even talk about ‘in kind.’ There’s only so many people we have that would be able to do this and we can’t - I’ve the term ‘in-kindedto death’ at various meetings. I think the blind spot for funding agencies are that they don’t realize there’s a cumulative effect of always asking for ‘in kind’”* (31).
Lack of appropriate organizational infrastructure	*“We really don’t have an opportunity for new relationships and new networks for partners to really get together… it’s still based on passive opportunities…. there isn’t really anything structurally in place, like having a central office of research…. [Research, evaluation, decision - support] has always been kind of ill-defined, it doesn’t really have a home. I think politically in part because we never really want to call things for what they are … the public [not wanting] to see money going into that kind of thing”* (23). *“[Before], academic researchers would basically knock at the door and would get ... a polite “thanks very much but we’re busy” and the door would close and that’s simply because the interface was not there”* (31).
Stresses on organizational staff working in liaison roles	*“It’s not a pretty place, to live in the gap. … On the academics’ side where merit promotion is captured, if you don’t have an open-minded department head, they’re going to say, and I have had times where they say to me, you need to focus, focus, focus and I’m like, mypaychequecomes from [the region], if I focus, focus, focus, I’m dead, I’m unemployed”* (05). *“We wanted to embed researchers into the healthcare system but no one thought it through… the implications of that for them as an academic”* (34). *“I’ve never encountered any resistance from my portfolio, the resistance I’m receiving is from the academic standpoint. Because it’s not aligning with what the real academic partners would want to focus on…What happens is they want someone to represent the subject matter experts within the clinical system … there is a lack of awareness of our role… but it could also be that if we don’t have an academic appointment, we’re not seen as on the same level playing field. ... until then they’re wondering, it could be – ‘why am I working in the system rather than working in academia?’”* (18).

Abbreviation: CEO, chief executive officer

However, many participants placed greater emphasis on systemic issues, particularly on the impact of health system restructuring and organizational stress. Health system restructuring and internal organizational change were noted as major challenges to partnerships by almost all. Changes were often observed to happen too fast and without supporting evidence; create dislocation and stress; and fail to support existing partnerships; resulting in a need to “*renegotiate what had already been achieved* ” (11). A change in leadership was observed to have significant positive or negative effects in a relatively short period of time (“*10 years and we* ’*re still waiting for the dust to settle down completely* ” (29)).

The impact of increasing healthcare demands at a time of shrinking resources was also highlighted, including the costs and additional stress resulting from expectations of research partnership. Several participants stated that they turned down requests to partner because they did not have the resources: the priority was patient care. Budgetary issues were identified as the main source of stress on those with senior leadership responsibilities – ‘keeping the doors open’ took precedence over attention to ‘evidence.’ Budgetary stress was described as resulting in both failure to provide organizational infrastructure to support partnerships (understaffing of research-related positions) and – because of the political pressure to cut administration and focus on patient care – in ‘hiding’ of investments in research roles rather than promoting their benefit. Another stress noted was that of leading and facilitating research partnerships, along with the specific challenges faced by those working in liaison research roles where mediating between different perspectives was both time-consuming and demanding of specific skills.

Another area of major challenge was identified as academic responsiveness and readiness to work in partnership and adapt to health system needs (Table 3). A mismatch between researcher interests and organizational needs was often observed, along with lack of researcher understanding of health system context. Many participants reported negative or frustrating interactions with researchers, although some qualified their response by stating that such experiences were rare, or had happened with only a few individuals. Concerns were raised about lack of respect from researchers; as well as a common inability to use appropriate methods in a practical setting; and limited preparation to work in partnership. A few participants, once reassured about confidentiality, shared issues that had created serious issues for their organizations.

**Table 3:** Academic Responsiveness and Readiness to Partner

Mismatch of researcher/health system interest with system needs	*“That’s definitely been the case… I have someone who’s very interested in doing research on X, which although is very interesting, researchers who are interested in working with the system are still looking to understand the problem, and we’re trying to respond to the problem. So sometimes, the research question is not in sync with service delivery needs”* (21). *“[Researchers] have their interest. And sometimes you have a question but you cannot find someone whose interest matches your need”* (29). *“What we more often find are researchers who have research interests that are not exactly aligned to those of the organization, to the organization’s needs. Then, collaboration is harder to establish…”* (13). *There’s always going to be a bit of a mismatch, because it depends on what the funders are funding, you know, the academic want to do”* (09).
Lack of understanding of health system context	*“Academia, it is focus, focus, focus. On the applied side, it broad, broad, broad”* (05). *“A misunderstanding or a lack of understanding of a) what research really is and what it includes, and b) how [researchers] can really make a difference or help in their day-to-day work”* (23). *“[Researchers] having an understanding of what is the reality of doing research and the stakes involved in research. We have certain constraints that we must respect”* (11). *“Most of the researchers don’t know that the health systems are dealing with these problems and they don’t know who to connect in with”* (36).
Lack of researcher skills in collaborative work	*“Some researchers ask me to help them, but they decide to move on without taking into consideration my recommendations. Then I have to tell them that their input is just one input among many in decision-making... there is always some friction”* (13). *“Academics are strong people and they worked hard for where they are.SoI think there are sometimes some kind of power imbalances... but then how it’s transitioned into a functional state with a health system to make that idea work, certainly that’s part of the relationship building and the give and take…. But there are some that want to see their idea applied kind of like as a whole. But when you put that into a very complicated and very messy system … there are modifications that need to be made.... Transitioning from an approved project to actually doing the project in the system, I think can get a little bit messy in there”* (23). *“Not all the time, but certainly it does happen, that the researchers are so focused on their agenda that they find the operating system one of frustration. ... I’ve actually had situations where researchers then figured out how to work around the system and have now got the system in an uproar because this is in fact breaching the law… ‘an environment of conflict vs. joint problem-solving’”* (08). *“I’ve had a couple of experiences where there’s very much a hierarchy, very, very symptomatic of a more traditional approach to research where one person is dictating what is required rather than really building a collaborative plan”* (21).
Inappropriateness of researcher behaviour	*“I’ve been involved in trying to manage and de-escalate a formal workplace disrespectful complaint. (The complaint was about a researcher) - the interaction became one that was completely unhealthy and to this day not able to manage constructively. I’ve also had to get a lawyer involved. The research is ongoing ... Now what we’ve got is basically an untenable working relationship”* (08). *“What we’ve heard is, that in a clinical domain, often the PhD students have no concept of what it’s like to be in a hospital, you know, and how you have to treat patients and how you have to have consent, and yeah, we have an incident right now which has kind of blown up in a small way.… a small explosion.… People are hearing about it now... we intervened to try and correct things but now it sounds like the patient is kind of taking things and running with it.… talking about their bad experiences to whatever audience he or she has”* (09).

While experiences with specific researchers were most often discussed, some participants differentiated between difficulties with individual researchers and challenges in working with universities: differences between universities in this regard were observed.

*“Every university, as you know, is different: different in their focus and different in are they easy to partner with ornot.* … *they are really bureaucratic and have all kinds of rules to the point where those working with them are just tired, you know.... sorry, if you* ’*re going to keep putting rules and boundaries and policies in place, we don* ’*t have time for it”* (35).

*“Well, you know, it depends on the university, because [University X] is in the catchment area ... I would say there* ’*s more sensitivity. Other universities that are elsewhere don* ’*t have [that] sensitivity, I would say. And, you know, again, I think it has to do with the complexity of large Health Authorities. It* ’*s harder to know who* ’*s who in the zoo”* (09).

Concerns were also expressed around other systemic issues. As described in Table 4, these included the requirements and limitations of research funding bodies; failure to provide inter-provincial and interregional supports for research partnerships “*For things like overhead and research support, so that we are able to financially and sustainably support that capacity to engage with academia* ” (31); and the rigidity of ethics review processes.

**Table 4:** Other Systemic Issues

Funder requirements	*“Sometimes the timelines are so unreasonable, and sometimes the release of calls is so, strategically I think, designed to manage volume…Timelines are not realistic”* (21). *“The model that is traditional for research is the Principal Investigator-driven organization of teams.… that model is not effective if the PI doesn’t have a good understanding outside of academia. It takes management skill, project management, partnership building, relationship management skills, other than just the research skills”* (26). *“There’s not tons of money for health services research, I would say. I mean, I look at a lot of the stuff that’s funded” (* 09).
Lack of government support	*“I think that government simply doesn’t understand the way academic health science organizations actually work on the ground”* (10). *“Difficulties we have… with government in making things happen. Impact of amalgamated regions (larger regions) on interest of politicians in getting involved in RHAs … we’re on the map everyday”* (29). *“We need evidence to inform and support teams at the federal level when it comes to discussion about, for example, funding and better supporting the healthcare system in terms of its need for things like overhead and research support, so that we are able to financially and sustainability support that capacity to engage with academia”* (31). *“There’s a bit of misunderstanding of where the health system is and how much more support it needs to do that kind of work because it [research] is not something the health system funds…”* (23). *“[In the government] veryhigh qualityperformers but no background in health and I think no common understanding about how research in health - what it is and how it could be used. [There is in X department] nobody who knows anything about acute care, which I found mind-boggling”* (1).
Failure to provide linkages between regions	*“Canada does not have an integrated health system and so we each all manage our own healthcare”* (08). *“There are pockets of great things but the system as a whole is not really primed to capture successes and generalizethem”* (23). *“It’s a barrier to know how to connect with people beyond your own network of relationships… We can’t shy away from the fact that a researcher in BC can inform needs in Nova Scotia”* (26). *“I think that it’s a big missed opportunity for the healthcare system because there is so much good work that is underway, and the only work that happens to be shared is through [accreditation] and leading practice reviews… for the most part it is word of mouth”* (36).

### An Evolving Context

In spite of challenges experienced with research partnership, several participants described partnership in research as evolving positively. As described in Table 5, some of this was attributed to initiatives of health research funders requiring partnership: Canadian health research funders, including the largest federal funder, the CIHR, have taken a leadership role in promoting a focus on knowledge translation and also developed several programs requiring health system partnership.

**Table 5:** Factors Contributing to Research/Health System Partnerships

**Key Themes**	**Sample Quotes**
Increasing interest in research by leadership within the health system	*“More and more ... they [health system managers] say they appreciate more and more research to support the paths they go down...they talk about needing to link up more kinds of academic activities and more of thatrigourto support decision-making”* (23). *“And I think we have, over the past five or 6 years, seen a great increase in the receptivity of our leadership in engaging in discussions to support research… We never would have seen this before where clinical leaders are absolutely embracing opportunities to collaborate with researchers”* (31).
Actions by research funders	*“Funders at every level want a better understanding on their investment in research and so, I think, the funding competitions have shifted in recent years, and are continuing to shift, to more application. I think the funders are moving in that direction and requiring it, so there’s a lot of it happening”* (10). *“This is kind of changing with the creation and supporting of grants around teams, and teams that are made up of different groups outside of the traditional health sector…the grant requirements are kind of forcing that...”* (23).
Increasing numbers of staff with research degrees	*“I think things have really improved, and part of that has nothing to do with us but because on the academic side, they’re turning out so many people with graduate degrees now. And now... it’s not just people with master’s degrees, its people with PhDs that are looking for work”* (09). *“We’re seeing more and more of PhD level [staff], as PhDs become less of a commodity. And I think there are a lot of people with PhDs who aren’t going to get an academic position”* (25).
Changes within academia	*“Academics, particularly in the health services area… they’re waking up to what are the priorities for the health authority... so there’s way more activity, there’s way more awareness. There’s people coming forward that want to develop acentrefor this or that”* (09). *“(About negative interactions). This is a problem that hopefully will not be around in the next like 5-10 years, because a lot of what I’m hearing, it’s the older academic physicians who have a set way of doing things and working that doesn’t always align well with what we need to be doing. But once they retire…”* (18).

Other factors noted were the increasing number of PhD and Masters prepared graduates employed by health regions; a generational shift leading to greater interest in collaborative approaches; greater awareness of health system priorities; and leadership actions taken by health regions themselves. There are some signs of a shift from simply ‘*approving or not approving* ’ (23) requests for research partnerships, to actively facilitating such partnerships and playing a developmental, mediating role.

*“When we explain the research world, which is unknown to them, they have greater empathy for researchers and the miscommunications that can happen. That is also true for researchers, to help them understand managers* ’ *issues”* (13).

At the same time, loss of a flagship CIHR partnership funding program (which required health system academic partnership) was noted, along with questions about funder support for continued partnership development. Other negative forces (unresolved data management issues; limitations on use of regional funds for research; and escalating budgetary and organizational stress) appear to be contributing to concern that these gains could be lost.

“*I’d like to think we’ve moved towards more integration, but if I use [what has happened in region X] as an example, if anything, some of that’s been lost”* (25).

### Strategies for Research Partnerships: Health Leadership Perspectives

Participants were asked about strategies they had found helpful in developing relationships with academic researchers, and suggestions they would give to other regions. As might be expected given the research-focused roles of many participants, a range of internal actions to promote and support partnerships were described and proposed. As described in Table 6, suggestions emphasized the need to develop positive and on-going relationships; to ensure strong leadership; to develop, clarify, and communicate conditions and processes for partnership; to ensure appropriate infrastructure to support partnerships; and to actively engage in activities to initiate partnerships. Although no interview question addressed the issue of leadership directly, the importance of leadership in creating a research-positive organizational culture, as well as in supporting effective research partnerships, was strongly emphasized.

**Table 6:** What Health Organizations Can Do

**Key Themes**	**Sample Quotes**
Develop positive, ongoing relationships	*“Relationship is key. Relationship, relationship, relationship. Getting to know each other, building trust, understanding where the challenges are for our partners in interfacing and having the same understanding for our clinical and health services people … and basically having all of the things that go into building a trusting relationship.… so being open, being transparent, sharing information, mobilizing knowledge, being able to resolve, to identify potential pinch points... That’s all soft skills”* (31). *“[Communication is] fundamental and must be regularly done”* (35). *“There’s such an opportunity to involve researchers upfront and help them understand the context that you’re developing policy within and the timelines that you’re working within because they can adapt and you can kind of adapt your methodology with them so they’re, you know, helping you along the way so you can iterate your strategy development”* (02).
Show strong leadership from the top of the organization	*“It all comes down to leadership. ... you need strong leadership with clear accountability”* (35). *“[The new CEO] brings the people definite stability, … definitely encouraging us to do more of an integration work, to create partnerships”* (18). *“I came to develop a vision of integrated research… so [this would not have happened] if the CEO had not seen thevalue”* (12). *“I’ve come to be a big believer that there has to be understanding from the most senior leadership within the organization. I don’t think I could emphasize that enough, that if the leadership doesn’t buy in, I don’t think there’s a chance of success”* (1).
Establish and communicate clear organizational processes	*“I think we need to be explicit about what a partnership or a collaboration looks like. I think organizations need to start to consider building in research evaluation into their business plan”* (25). *“Lay out your expectations explicitly at the start of a partnership. … defining how you expect the researchers to engage with you… talking about what are issues you anticipate could occur and how will you mitigate those”* (04). *“They may not realize it, but unless they can get our buy-in, the [research projects] are not going to be approved. They may not realize that and they may be really angry about it”* (18). *“[Previously] the academic person would kind of come into [the region] and - this is the story I was told - kind of terrifying people into having them do what they wanted…. So [now] we say to the institutions: your researcher can come here and play in our sandbox. Here are our rules.Soit keeps everyone kind of honest”* (09).
Develop infrastructure to support partnerships	*“Sowe have also hired at [X], organizational researchers. These are folks we ask to do research, like any other researcher. They don’t have teaching obligations, obviously, they are paid for by the organization. We don’t expect them to apply for salary grants, but we do ask them to submit research projects to funding agencies and align their research program to the organization’s priorities and needs” (13).* *“Now we have certain structures in place to involve academics, for instance we have a Primary Health Research Network. And through that venue, and through a community of practice that we’re starting, it’s where we communicate to say ‘here is what the burning issues are on the operations side, and if you’d really like our support, stand behind us’”* (05).

Abbreviation: CEO, chief executive officer.

Many discussed actions the region had taken, or would propose, in order to increase regional appreciation of the value and potential contribution of research, and several gave examples of criteria they had established to better respond to and manage research relationships. The most commonly cited criteria for partnership was a fit with regional priorities. Other common requirements included: impact/access review of resource demands; involvement and approval from relevant departments; and requirement that any costs (eg, data extraction) be covered. Reflecting common observations on the lack of infrastructure to support academic/organizational research partnerships, several participants highlighted the need for health organizations to invest in research roles and take clear action to promote effective partnerships. Two main models of internal regional restructuring to better support research partnerships were discussed. The interface approach focused on creating spaces to encourage, support or “force” collaboration by providing settings, (such as networks or bodies that provide a ‘table’ for academics and health partners to develop common agendas) where research partnerships could be identified and nurtured. The embedded expertise model, in contrast, focused on building internal capacity through hiring organizational researchers or investing in specialized research roles such as knowledge or relationship brokers. Several regions had adopted more than one approach, and it was observed that: “*There* ’*s no one strategy, it* ’*s a bucket of strategies* ” (05). Some participants discussed the advantages and disadvantages of these models. Many preferred the interface model given the limitations of regional resources, recognition of university-based expertise, and constraints in some regions against using organizational funds for research purposes. However, there was concern about relying too heavily on an external body to meet regional needs (“*we may be a bit naive about having an entire dependency on another institution to support research within [the region]* (17)”). Some expressed lack of confidence in the preparation university-based researchers received to work with regions, and academic ability to respond to specific regional needs and time frames. There was concern that academic incentives may discourage researchers from doing effective partnership research: as one participant observed: “*you have to know who your master is* ” (05).

Some participants referred to specific initiatives to promote and support partnership research of interest to the region. These included participation in post-doctoral training initiatives, a researcher-in-residence role,^62^ an affiliated researcher program with the university for in-house researchers, working through communities of practice, matching services (between academic centres and regions or regional programs), various forms of research days with universities (where parties “*pitch their research ideas to each other* ”*)* (09), and seed funding for collaborative research operated by the region/province. However, while recognizing the need for organizational initiative, participants placed significant emphasis on changes they felt were needed within other systems.

As described in Table 7, participants strongly suggested that multi-system changes are required. Some stated a need to ‘re-imagine’ research: how it was defined, how it related to other knowledge-generating activities, and how research partnerships were framed. Some referred to the need for institutional level agreements (university-health region), not simply collaborations between individual researchers and managers. Others commented on the need for leadership both within academia and at the provincial level.

**Table 7:** A Call for Multi-system Change

**Key Themes**	**Sample Quotes**
A need to re-imagine research	*“There needs to be a reimagining of how you engage the sector in research”* (21). *“I think there needs to be that re-imagination.… this can’t be seen as something distinct from QI... but how do you make it an integral part of the work?SoI think they’ve really got to relook at their work through a different lens. … I believe there has to be ongoing and regular communication between universities and academics with the universities and the senior leadership both within regions and within governments… to develop these relationships and sustain them”* (01). *“Creating a culture of learning and innovation… ‘a way of approaching things.’ I get the need to have acentreof excellence, but at the end of the day, we need this to become the way we do work … Research, evaluation, innovation need to build into our system so that is at the heart of it and it feeds everything. As opposed to something you parachute in”* (25).
Moving beyond an ‘acute care’ and clinical focus in research	*“The research that you hear about is really about needing the foundations to raise money… for children’s programs and things like that. That’s kind of like, those are the okay places to do research, where the not so okay places are in public health and community and decision-making and things like that”* (23). *“There’s this kind of divide, right. There’s clinical research and then there’s community research or program evaluation or program development. And I would like to see that soften a bit - just be more of an open market around funding evaluation and research…”* (25). *“When you realize that about 90% of healthcare it’s within the community….likewe’re missing a major, major, major piece”* (35). *“Governments really care about a very small part of the health system. … It’s a small part of what ahealth systemsis and delivers”* (01).
Rethinking research funding	*“The grants are not tailored for the community servicepartners,they always require some specific Principal Investigator who has an academic affiliation”* (18). *“Need training for peer reviewers … looking at peer review practices, moving away from the traditional peer review to the broader peer review that encompasses patients, encompasses policy-makers, other relevant stakeholder groups”* (26). *“We get asked for hundreds of letters of support… it’s researchers looking to get a perspective of being seen as collaborative, and wanting to strengthen their application. But often there’s no flow of resources”* (25). *“And a much more open collaborative approach and a recalibration…. not just getting more funding to the select few with a strong research portfolio. I think restructuring at that level is needed”* (21).
Improving academic preparation for health services research partnerships	*“It’s mostly the academics who want to partner, that are really thinking of specific projects they want to partner on, and they just want money for those. Are they prepared to work with healthcare services? I don’t think they have that training to come in with”* (18). *“The need for leadership training for academics... such as LEAD - or a form of leadership development that equips them with the skills of understanding the issues related to system complexity, so they can get thathigh levelview of how and where their research fits into this big moving target”* (31). *“Researchers having, if they don’t already, training on how to enter communities and work with partners with that kind of humility that I mentioned… ensuring that researchers understand the different contexts that they are working in and the factors that could influence their success and their impacts on communities – being community in the large sense, including health systems, etc. Some maybe need some kind of crash course in the context... and training before they can understand what it means to communicate in that respectful way”* (04).

Abbreviation: QI, quality improvement.

*“The previous minister of health in this province had a pretty big influence for several years and [s/he] had almost no use for research that wasn* ’*t directly producing results in the clinical area or producing innovation in commercialization. The new minister is much morefavourable* … *But it* ’*s true that the politics and the perspectives of leaders in the system does have an impact that can last for several years before it can be shifted”* (17).

While many recognized the role that research funders had played in promoting research partnership, some stated that “*more needs to be done* ” (05) to support and require authentic partnerships. Some were concerned that partnership continues to be driven by a call for proposals, not necessarily organizational needs. There were calls to ‘*level the playing field.* ’ with several expressing concern about the balance of research resources for community vs acute care; others called for more support for community-based research, for evaluation research and to “*address the more administrative questions* ” (29).Participants identified a need for training for peer reviewers, and examining current posting and peer review practices to include the perspectives of “*relevant stakeholder groups* ” (26). It was proposed that review processes be revised to support authentic partnerships. The need to support infrastructure within regions to facilitate research partnerships and better respond to system research priorities and time frames was emphasized; it was felt that regions should be adequately compensated for their contributions to research.

Many suggestions were made about academic training, which was often viewed as inadequate for preparing researchers to work collaboratively with health organizations, and as divorced from the realities of the healthcare system. In addition to identifying gaps in training (as discussed under challenges), some participants also emphasized the need for ‘soft skills’ and leadership training.

Some participants also commented on the need to address perceived rigidity in ethics related to health services research, noting that unnecessary bureaucracy and artificial boundaries between QI and research often prevented the health system from incorporating the expertise of researchers.

## Discussion

This study, one of the first to examine in-depth the experience of health system partners with academic research partnerships, provides important insights into how research is defined and understood with the Canadian health system, as well as how research partnerships have been experienced. Health system participants identified additional challenges to research partnerships to those previous identified in the literature. Findings suggest that significant changes are needed both in how we think about the concept of ‘research’ as it contributes to health system design and health services organization, and in actions needed to promote effective research partnerships.

### Reimagining Research: Making Research Relevant

As discussed in the results section, a major emerging theme was the call to ‘reimagine’ research and our assumptions about research partnerships if research is to make the same kinds of contributions to questions around healthcare organization as it has made in the bench science or clinical realm. Participants propose that understandings and definitions of research should evolve to encompass the potential (and mutually beneficial) overlap of research with other forms of organizational learning (such as QI and evaluation).^63,64^ The blurring of boundaries between research and its use facilitated by research partnerships^65^ would encourage greater creativity in integrating research expertise into the fast-changed pace of healthcare improvement; promote more rigorous evaluation research^66^ and address the common separation of research, evaluation, and QI.

The complex and fast-paced environment in which health services/systems research must take place, combined with a lack of appropriate preparation and support for academics to assume helpful partnership roles, requires multi-system action: challenges cannot be addressed by individual researchers or by health organizations working alone. This need for collaborative approaches across sectors, which has been highlighted in the policy field, should be further developed within the area of health services/system design.^67-69^

Response to the often-repeated calls to address the mismatch between academic preparation and interests and the realities of health system improvement has been slow.^70^ If researchers are going to contribute to health system transformation and assist in development of a culture of learning and improvement in health organizations, they require practical training and mentoring in partnership research^34,54^: this preparation should be directed by the needs and insights of the health system itself. Joint development (and evaluation) of actions could be a first step in addressing the attitudinal issues (lack of ‘humility’) that too often present challenges to true partnership.

Research funders and provincial governments have a critically important role. Responding to system experience may enable research funders to better support health system transformation. First, changes to the proposal review process (ensuring health system expertise has a clear voice in the review process; requiring teams to include research leadership with system credibility; valuing members with partnership skills) could promote greater accountability to health system priorities. Encouraging organizational (university/health organization) linkages rather than relying only on researcher/manager relationships could also promote greater academic accountability. Specific actions to address the mismatch of funding timelines with health system needs (eg, continuous intake, rapid review, simplifying decision-maker application requirements); to promote development of effective partnerships (eg, meeting and planning grants)^44^; and to provide appropriate organizational financial support (allowing organizations to find ‘time’ and resources to partner)^39,54^ would also facilitate partnership development.

The mismatch of ethical requirements for both health services research^71-73^ and collaborative approaches^74^ has been documented. These ethical requirements, developed in response to the need to protect individuals from experimental procedures, may often (because requirements are viewed as onerous, time-consuming, slow and inappropriate) lead to avoidance of research activities, or branding them as ‘QI’ or ‘evaluation’ to avoid the requirement of ethical review.

Our findings also challenge some assumptions about barriers to, and facilitators of, research partnerships. Findings that healthcare decision-makers often do not find research timely or relevant are not new,^19,20,65^ nor is the emphasis on the importance of establishing respectful and trusting relationships between researchers and knowledge users.^75^ However, it is necessary to move beyond standard descriptions of barriers.^50^ While participants identified communication issues (a commonly-identified challenge) as an issue, many felt that these were not so much a result of differences in language and culture as a failure to listen to each other. Sometimes, communication problems occurred because the expertise and insights of health system personnel were not taken into account – that the ‘humility’ and respect for the diverse perspectives needed to address complex problems^76^was missing. This suggests that efforts to train researchers simply to better communicate findings (rather than how to work in partnership) and to train health system personnel to better appreciate and understand research may be misplaced.

Participants described leadership commitment to enabling research as an essential pre-condition for research involvement, highlighting the challenges of health system stress and healthcare restructuring. These issues have received less attention in the literature: we found only a few articles addressing the issues of leadership or structural change (except as how personnel change affects relationships between specific researchers and decision-makers).^20,47,65,77^

Nor are some commonly-held assumptions about the role and contribution of academics shared. While the literature emphasizes the risks to young researchers of collaborative research earlier in their career,^8^ this is in contrast to the benefits of collaboration to younger researchers highlighted by some participants. Assumptions that ‘rigour’ comes from academia and ‘relevance’ from the system were also challenged, as participants gave examples where methods proposed were not appropriate for the research questions.

### Future Work

To date, little attention has been directed to the experience of health system leadership with research and research partnerships, or to evaluating various strategies for supporting partnerships between academics and the health system. Our findings suggest that significant changes may be needed to how health research is defined and promoted if the research benefits achieved in the basic and clinical science field are to be achieved in the field of health systems/services research. Many potential strategies for improvement have been proposed. While there is increasing interest in creating “embedded” positions, and emerging evidence on potential benefits of such positions,^78^ ‘embeddednesss’ is a concept with many different interpretations.^73,78,79^ Evaluation of the benefits and disadvantages of various expressions of these roles (eg, researchers hired by health organizations; types of research and evaluation units; researcher in residence roles; knowledge brokering) is needed. At the same time, evaluation of strategies for enhancing researcher readiness for health service/system work, as well as for development of ‘spaces’ at the interface of these 2 systems^23,80^ would be beneficial.

### Strengths and Limitations

This research is one of the first studies focused on the perspectives and experiences of Canadian health leadership with health organization-university researcher partnerships. Although limited by the number of participants, this national study is one of the few including the province of Quebec. Perspectives cannot be assumed to be reflective of all Canadian health leadership, however, as sample selection focused on regions with demonstrated research activity, and where interest had been identified. Nor can it be assumed that the perspectives of the individuals interviewed were representative of all leadership within their region: those interviewed were often in ‘hybrid’ positions linking research and operations. This sampling approach can be expected to result in overestimation of research awareness and interest. Some regions were unsampled: in 2 provinces, research ethics requirements created barriers to our requests for an interview; in other regions, a research contact was not apparent, and there was no response to our attempts to identify and contact someone who could speak to the issue. It may be that these regions have less developed research partnership responses. Social desirability bias may also have affected participants’ responses, particularly as the sample was selected based on evidence of leadership or regional interest in the topic, and it was noted that many participants were eager to discuss accomplishments of their organization. Some participants also expressed concern about ensuring anonymity of responses before sharing less than positive experiences, which also suggested that, in some cases, social desirability bias may have been an issue.

While the Canadian health system may differ from that of other countries, the dearth of evidence on academic/health system partnerships focused on health system design and health service organization (in general), and on the perspectives of senior health managers on such endeavours (in particular) suggests that there may be a need in all jurisdictions to investigate the perspectives and experiences of health system leaders and managers. Regardless of the particular type of health system structure, effective partnerships rely on relationships between researchers and knowledge users that are collaborative, involved, and meaningful.^68^ All systems will benefit from asking about and understanding the perspectives of senior managers with their health systems, which may be very different from academic perspectives. While re-imagined research may express itself differently in various jurisdictions (and further contributions could explore context-relevant approaches), key principles for productive partnerships (eg, humility, respect) are likely to be applicable in all settings.

## Conclusion

New ways of supporting research partnerships between health organizations and academia are required, particularly in the field of health service/systems research, if research is to make needed contributions to the many challenges facing healthcare organizations. Effective action to promote and support research partnerships in the fast-paced context of today’s healthcare system will address the separation of research from healthcare management and support development of health organizations that promote a culture of learning. There is a limit to the progress health regions can make without the engagement of provincial health departments, research funders, and academic institutions.

## Acknowledgements

This research was supported by the CIHR funded Foundation Grant (FDN #142337) “Moving Knowledge into Action for more effective practice, programs and policy: A research program focusing on integrated knowledge.”

## Ethical issues

Ethical approval for the research was received from the University of Manitoba Health Research Ethics Board.

## Competing interests

Authors declare that they have no competing interests.

## Authors’ contributions

SB: Conception and design, data collection, data analysis and interpretation, first draft of article. IB: Principal Investigator, Funding acquisition, conception and design, data collection, data analysis and interpretation, critical review, supervision. IDG: Funding acquisition, conception and design, interpretation, critical review. MM: Conception and design, data interpretation, critical review. DdM: Conception and design, Data collection, data interpretation, translation, critical review. KH: Conception and design, data interpretation, critical review. BL: Data interpretation, Critical revision. CU: Data interpretation, critical revision. JK: Critical revision.

## Authors’ affiliations

^1^Applied Research and Evaluation Consultant, Centreville, NS, Canada. ^2^Department of Community Health Sciences, University of Manitoba Winnipeg, Winnipeg, MB, Canada. ^3^Ottawa Research Institute, University of Ottawa, Ottawa, ON, Canada. ^4^School of Nursing, University of Northern British Columbia, Prince George, BC, Canada. ^5^Universite de Saint-Boniface, Winnipeg, MB, Canada. ^6^University of Winnipeg, Winnipeg, MB, Canada. ^7^Hôpital Montfort, University of Ottawa, Ottawa, ON, Canada. ^8^Northern Health, Prince George, BC, Canada. ^9^University of Northern British Columbia, Prince George, BC, Canada. ^10^Nova Scotia Health Authority, Halifax, NS, Canada.

## Supplementary files


Supplementary file 1 contains list of challenges presented to participants.

## Key messages

**Implications for policy makers**

- Exclusion of the insights and experiences of health system leadership has limited the effectiveness of many initiatives designed to promote greater use of research and participation in research partnerships in the rapidly evolving healthcare environment.
- Definitions of research must be broadened to encompass the range of activities (including quality improvement [QI] and evaluation) associated with health system learning if the full benefits of research are to be achieved. For this to occur, the many barriers to integrating research into the fast-paced healthcare context must be addressed.
- Improvements are needed to educational programs for health services/systems researchers. Universities should focus on preparing researchers to understand the complexity of health service organizations, use a broad range of methods, and develop the interpersonal skills and attitudes necessary for meaningful multi-sector engagement.
- Research funders should continue to review and evaluate current funding programs to ensure that health system research priorities can be responded to in a timely fashion; that authentic partnerships are recognized and valued; and that review panels reflect the breadth of skills needed to evaluate proposals.
- For effective research partnerships, health organizations must demonstrate strong committed leadership in this area; commit to development of ongoing research relationships; establish clear criteria and processes for collaboration; and invest in the infrastructure required to ensure active health system participation in research activities.

**Implications for public**

All countries are searching for ways to provide the highest quality patient care. A promising strategy to do this is to require researchers to work collaboratively with health system personnel to ensure that the most important questions about how to deliver health services are studied, and that research findings are integrated into practice.

This research, which explored the experience of senior personnel within health organizations with research partnerships, found that they often find research unhelpful or irrelevant. Improving the training of health researchers, making changes to how research is funded, and concrete actions to integrate research skills into quality improvement (QI) initiatives, could accelerate the health system learning needed for citizens to obtain full benefit from investments in health research. These changes to promote effective research partnerships require action not only by health organizations but also by universities and by research funders.
