# Supplementary material for: Experience of Health Leadership in Partnering With University-Based Researchers in Canada– A Call to "Re-imagine" Research
Source: Int J Health Policy Manag. 2019 Aug 7;8(12):684–99. doi: 10.15171/ijhpm.2019.66 (PMC6885864; doi:10.15171/ijhpm.2019.66)
Supplement: Supplementary file 1 — contains list of challenges presented to participants. [file ijhpm-8-684-s001.pdf]

Supplementary file 1.

List of Challenges Presented to Participants

Has (your organization) ever experienced any of the following challenges or difficulties related to development and management of partnerships with academic (university-based) researchers?

__ Didn't know how to find researchers with the expertise you needed?
__ Weren't sure how research could help?
__ Regional timelines for action didn't allow for research partnership development?
__ Researcher interests were not priorities for the region?
__ Research requirements were not realistic?
__ Costs of research to the region were not recognized?
__ The region did not have internal expertise?
__ Mismatch of agendas between region and academia?
__ Problems related to communication?
__ Negative interactions with researchers?
__ Structural changes within your organization?
__ Any other challenges that I did not mention?
